# COMPARATIVE ANALYSIS OF FALL RATES AMONG COMMUNITY-OLDER ADULTS IN SIX COUNTRIES

**DOI:** 10.1093/geroni/igae098.3941

**Published:** 2024-12-31

**Authors:** Hadi Kooshiar

**Affiliations:** Texas A&M University-Corpus Christi, Mission Viejo, California, United States

## Abstract

The number of older people who desire to live in their homes is rising. So, this study aimed to evaluate fall rates in community-dwelling older adults across six English-speaking countries. This cross-sectional survey was conducted with 114 older adults residing in Canada, the USA, Australia, Ireland, New Zealand, the UK, and Northern Ireland. Participants recruited online through social media and passive snowball sampling completed self-rated fall risk (FRQ) and Activities-specific Balance Confidence (ABC-6) surveys, along with sociodemographic and health-related questions. Canadian and Irish respondents comprised the highest and lowest proportions of the sample, at 37% and 9%, respectively. The mean age was 67 (SD = 7.8), with a balanced gender distribution. Chronic diseases, notably joint pain and abnormal blood pressure, were reported by 25.4% of respondents. 44.8% of respondents reported falls in the past year, with 35.1% reporting falls in the past six months. Multiple falls were reported by 25.2% of respondents, with 45.8% reporting fractures primarily in the hands, forearms, or arms. Fall rates varied significantly among the countries: Canada (54.1%), USA (44.4%), Australia (53.8%), Ireland (40%), New Zealand (41.7%), and the UK and Northern Ireland (23.5%). Geographic, demographic, and methodological factors influence these rates. Higher fall rates in Canada may be due to an aging population and adverse weather conditions. The study’s findings have direct implications for research, clinical practice, and education. Recommendations for a prospective design with a large sample size are essential for a more comprehensive understanding of falls rate and effective prevention of falls.

